# Modeling of Polyolefin–Aluminum Bonding Technology Under Electromagnetic Energy: Using Hot-Melt Adhesives with Metallic Micro-Additives

**DOI:** 10.3390/polym18080930

**Published:** 2026-04-10

**Authors:** Romeo Cristian Ciobanu, Radu Florin Damian, Mihaela Aradoaei, Cristina Mihaela Schreiner, Alina Ruxandra Caramitu, George Ursache

**Affiliations:** 1 Department of Electrical Measurements and Materials, Gheorghe Asachi Technical University, Bdul. D. Mangeron 71, 700050 Iasi, Romania; rdamian@etti.tuiasi.ro (R.F.D.); mosneagum@yahoo.com (M.A.); cschrein@tuiasi.ro (C.M.S.); george.ursache@gmail.com (G.U.); 2 National Institute for Research and Development in Electrical Engineering (ICPE-CA), 030138 Bucharest, Romania; alina.caramitu@icpe-ca.ro

**Keywords:** polyolefin/aluminum bonding technology, hot-melt adhesives, metallic insertions, electromagnetic energy, GHz range

## Abstract

Polyolefin bonding technologies with metal foils are extensively employed in various sectors, particularly in automotive, electronics, and aerospace industries. This research examined the innovative electromagnetic joining of polyolefins to aluminum by evaluating the behavior of hot-melt adhesives derived from polyolefins containing metallic particles. The study aimed at establishing the specific absorption rate (SAR, expressed in W/kg) via electromagnetic simulation using CST Studio Suite software. It was observed that, regardless of particle size, Al was the most efficient particle, while the distribution of particles has a negligible impact on Total SAR values. The most significant beneficial effect of the inserts on the absorption capacity of the hot-melt material is primarily observed with a particle size of 1 μm. When connecting polyolefins to aluminum, the power loss density and SAR values exceed those for bonding polyolefins to polyolefins by at least 10 times, owing to aluminum’s conductive properties, which influence the absorption of additional energy in the hot melt mass, likely due to the Salisbury screen effect generated by the bonding arrangement. For hot melts made from polyethylene, a higher frequency of 5.8 GHz is suggested, which is a newly approved frequency used in advanced industrial applications. This positively impacts the effectiveness and viability of the bonding process of polyolefins to aluminum, resulting in reduced exposure times and/or decreased microwave exposure power. It was observed that the hot melts derived from HDPE and PP yielded greater SAR values. Conversely, the SAR values increase when aluminum is attached to HDPE. As a result, the strongest bond of polyolefins to Al occurs when connecting HDPE to Al using HDPE-based hot melts. The proposed simulation methodology may offer considerable improvement in evaluating the efficacy of bonding technology for dissimilar materials subjected to electromagnetic energy

## 1. Introduction

Hot-melt bonding methods are of extreme economic interest and have a significant market influence (the hot-melt adhesives sector was valued at USD 8.29 billion in 2025 and is expected to increase from USD 8.70 billion in 2026 to USD 12.99 billion by 2034, showing a CAGR of 5.20% throughout the forecast period [1]).

Hot-melt adhesives, which are glue types based on polymers, are used when they are melted, transitioning from liquid form at elevated temperatures to solid form at cooler temperatures within a narrow temperature range. After the hot melt cools, it solidifies and forms an adhesive bond between the two chosen surfaces [2]. Hot-melt adhesives offer significant advantages for large-scale production, as they come with low costs thanks to their easy application, rapid bonding, and utilization of a single component without requiring extra materials or catalysts for adhesion. Electromagnetic field-activated adhesive bonding technology is pioneering and serves as an alternative to the thermal bonding methods currently utilized in manufacturing industries. The primary benefit is the consistent heating of the hot melt throughout the entire volume before the bonding surfaces are heated, ensuring the hot melt achieves adequate temperatures without significantly affecting substrates, thereby avoiding overheating and mechanical residual stresses.

Bonding technologies of polyolefins and their composites (of low and/or high-density polyethylene—LDPE/HDPE, or polypropylene—PP) with metal foils are widely utilized across different industries, especially in fields such as automotive, electronics, and aerospace. Although hot-melt bonding methods are frequently employed in multiple technological contexts to connect different material surfaces in various setups, successfully bonding polyolefins with aluminum can pose technological challenges because of the low surface energy of polyolefins and low reactivity of aluminum to classical hot melts. In particular, for polyolefins, various hot melts were evaluated, yet their application required either a particular co-polymer [3,4] or prior treatment of the bonding surfaces, such as Corona, flame, or plasma treatments to increase the surface energy of polyolefins [5,6,7]. Currently, specialized adhesives are required to bond polyolefins to aluminum, in addition to treating the surface of the polyolefins. Efficient techniques involve utilizing Methyl Methacrylate (MMA) adhesives, structural epoxies, or surface activation (plasma/flame/primer) paired with cyanoacrylates [8,9,10,11]. To achieve stronger, long-lasting joints, it is essential to use additional specialized primers for the polyolefins [12,13]. On the other hand, the aluminum surface must be cleaned, degreased using a solvent such as isopropyl alcohol, and preferably abraded or chemically treated (e.g., anodized) to eliminate the weak oxide layer and enhance adhesion. In recent years, functionalized hot-melt adhesives based on polyolefins, especially those incorporating ethylene–vinyl acetate copolymers or maleic anhydride-grafted polyolefins were used, providing stronger adhesion at temperatures of 160–190 °C [14,15]. However, in all cases described above, delamination and partial separation of layers often occur.

This paper focuses on the advancement of hot-melt adhesives made from polyolefins with metallic ingredients for the bonding of polyolefins with aluminum. The quantity of heat required for bonding under electromagnetic energy is influenced by the characteristics, proportion, and shape of the metallic particles, particularly due to the dielectric characteristics of composite hot melts in the GHz frequency range [16,17,18,19,20,21].

Different materials have been researched as inserts in dielectrics due to their current application in microwave absorber technology [22,23,24] or for their impact on electromagnetic energy [25,26,27], particularly by enhancing absorption in the microwave frequency range. In previous studies of the authors [28,29], a reversible joining technology for polyolefins using electromagnetic energy and homologous hot-melt adhesives containing metallic and ferrite additives was successfully simulated and technically demonstrated.

Currently, numerous studies focus on the development of materials for electromagnetic interference shielding, yet there are comparatively only a few electromagnetic modeling efforts and simulation models involving interference shielding features of composites, including nano/micro-materials [18,19,20,21]. In all electromagnetic applications, including our case of polyolefin/aluminum bonding technology under electromagnetic energy using hot-melt adhesives with metallic additives, initial simulations of dielectric properties of joints are necessary for improved experimental design before accurate technological tests upon relevant samples can be performed because of the expensive nano/micro-materials, technologies, and testing methods.

This study consists of a new approach of modeling that uses a specialized software (CST Studio Suite—Electromagnetic Field Simulation Software [30]) of polyolefin/aluminum bonding technology under electromagnetic energy using hot-melt adhesives with ingredients that optimally absorb electromagnetic energy in the GHz frequency range. The innovation of this paper is related to the use of similar hot-melt adhesives with metallic additives for polyolefin/aluminum bonding technology, assuming that these types of hot melts could enhance their physical–chemical affinity to both the polyolefin substrate and the aluminum foil. To our knowledge, this research is the first to demonstrate the potential of using polyolefin-based hot-melt adhesives with metallic additives for the bonding technology of polyolefins/aluminum through microwave energy and may open new technological opportunities for applications across different sectors, especially in automotive, electronics, and aerospace fields.

## 2. Materials and Methods

### 2.1. Materials

The metallic inserts must be introduced in a polymer matrix made of polyolefins (LDPE/HDPE, PP) to generate a hot melt, but for polyolefins, the CST Studio Suite software lacks models in its library. Consequently, a new framework was developed utilizing the 1st-order Debye Equation [18]. The results are presented in Table 1.

Concerning the metal inserts (iron and aluminum), the software features predefined models within libraries, as shown in Table 2. Aside from their electromagnetic properties for facilitating the technological heating of composites with microwave energy, iron and aluminum particles were chosen due to their stability, affordability, and broad availability, making them suitable for mass production for hot melt applications.

### 2.2. Modeling Method

When dealing with low-loss materials, the measurement inaccuracies significantly impact the precision of the results and deviate from the classical models of the study of the interaction of the electromagnetic field with matter. In our case, the electromagnetic simulation relies on the method initially suggested by Nicolson, Ross, and Weir (NRW procedure), adapted as demonstrated, for instance, in [31], to address the NRW procedure’s instability. The NRW technique offers a precise, direct computation of the permittivity and the reflection and transmission parameters, more accurately assessing the electromagnetic energy absorbed by materials. This method seeks to substitute a dielectric material with inserts for a comparable material regarding its performance in the electromagnetic field, with excitation occurring as a plane wave. Volumetric energy losses (W/m^3^) can be directly assessed when analyzing polymeric materials containing metal inserts, since the sole heat source comes from the dissipation of electromagnetic energy within the structure. The main electromagnetic analysis via CST Studio Suite software [30] relies on the computed electromagnetic field values within the composite structure, as detailed, for instance, in [18,32], where the cell model was defined as a parallelepiped structure and boundary conditions were also established.

The studies aimed at identifying the specific absorption rate (*SAR*, expressed in W/kg), which is defined as the time derivative of the energy (*dW*) lost in the mass unit (*dm*) inside the volume unit (*dV*) of given density (*ρ*), (1), [32].

(1) $$SAR=\frac{d}{dt}\left(\frac{dW}{dm}\right)=\frac{d}{dt}\left(\frac{dW}{\rho \cdot dV}\right)$$

SAR assessment is provided in CST software as an additional step after the primary electromagnetic simulation, utilizing the calculated values for the electric field. Usually, the SAR results are calculated by averaging over standard elementary masses (such as 1 g); however, in this case, the averaging was conducted using lesser masses (as small as 10^−6^ g to analyze finer details of the inserts).

The simulation presented below for hot-melt composites with metallic additives is groundbreaking and seeks to identify the best choices related to particle size and power loss at different frequencies, serving as a foundation for advancing bonding technology in the microwave domain.

The modeling becomes more precise when accounting for quasi-cubic/parallelepipedal particles, equating volumes of dissimilar shapes, as it is anticipated that electromagnetic energy losses in metallic particles are superficial rather than volumetric. Consequently, in each subsequent stage of the simulation, parallelepiped-shaped particles equivalent to spheres were considered, with the mathematical equating approach extensively detailed in [33,34]. To address the identified inconsistencies in the dimensions of the nanoparticles, we permitted each side of every cube to vary between half and double the standard cubical edge, leading to a randomly shaped parallelepiped particle that is also randomly positioned.

**Figure 1:**
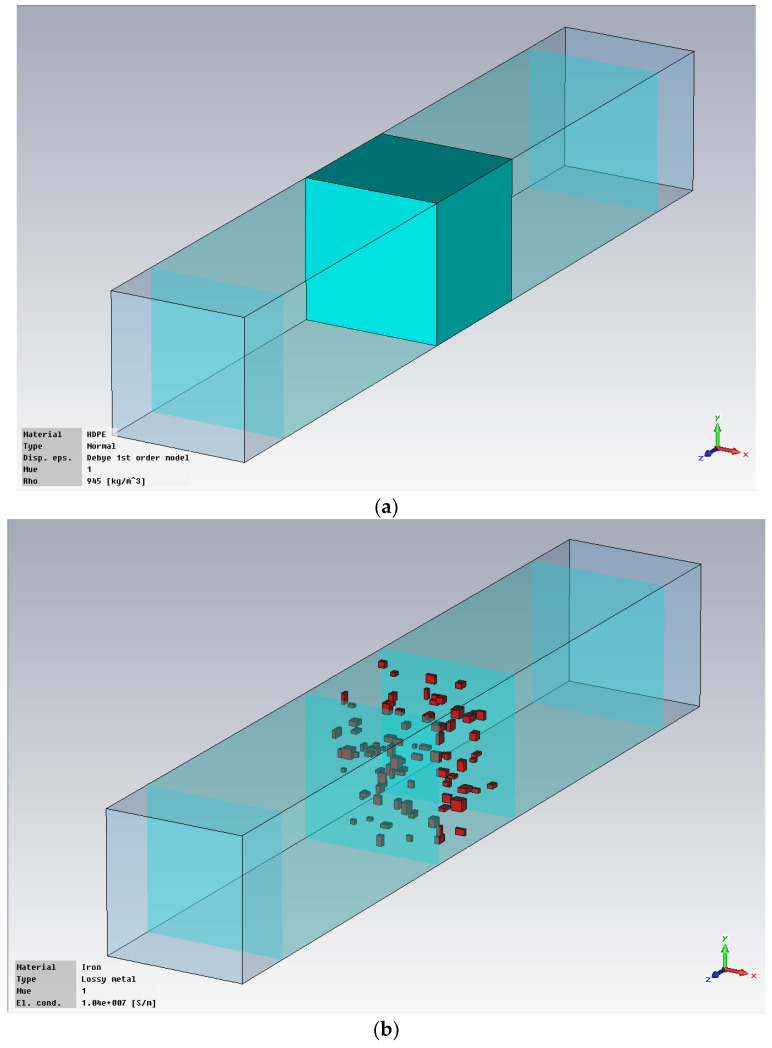
Modeling of hot-melt interlayer: (**a**) polyolefin matrix; (**b**) metallic inserts.

**Figure 2:**
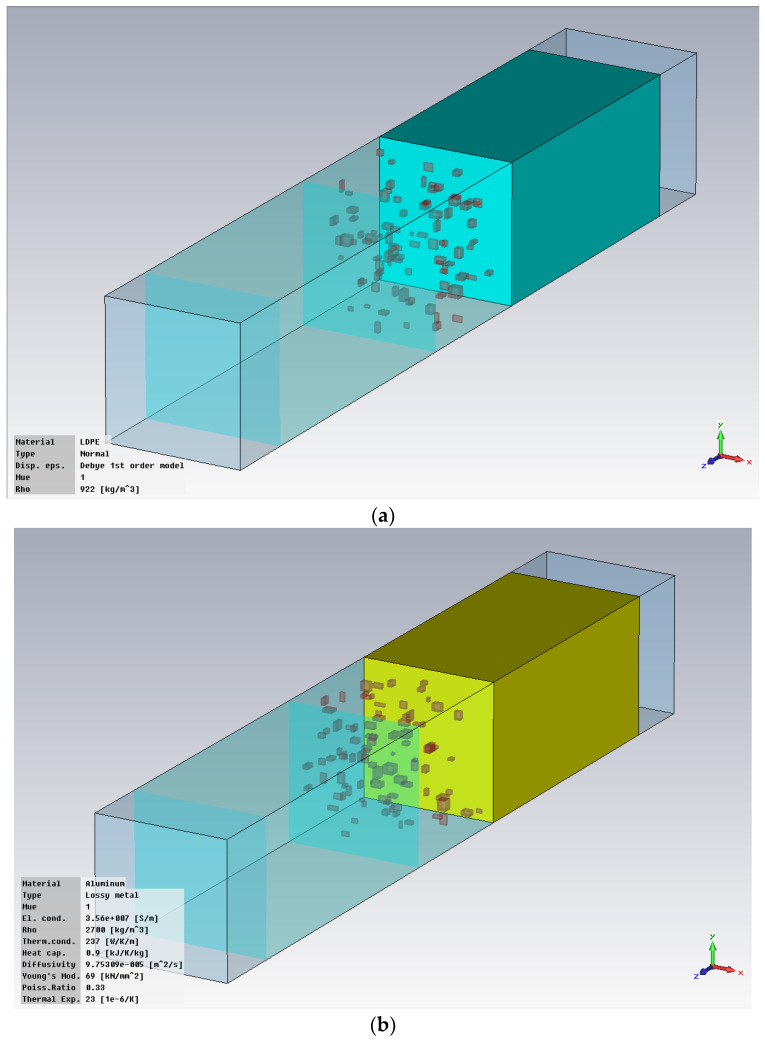
Modeling of the junction between one substrate and hot-melt interlayer: (**a**) polyolefin with metallic inserts; (**b**) Al.

**Figure 3:**
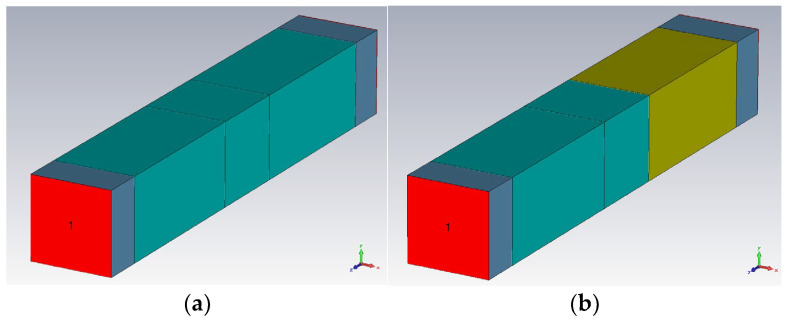
Modeling of the full structure of bonded material: (**a**) polyolefin to polyolefin; (**b**) polyolefin to Al.

**Figure 4:**
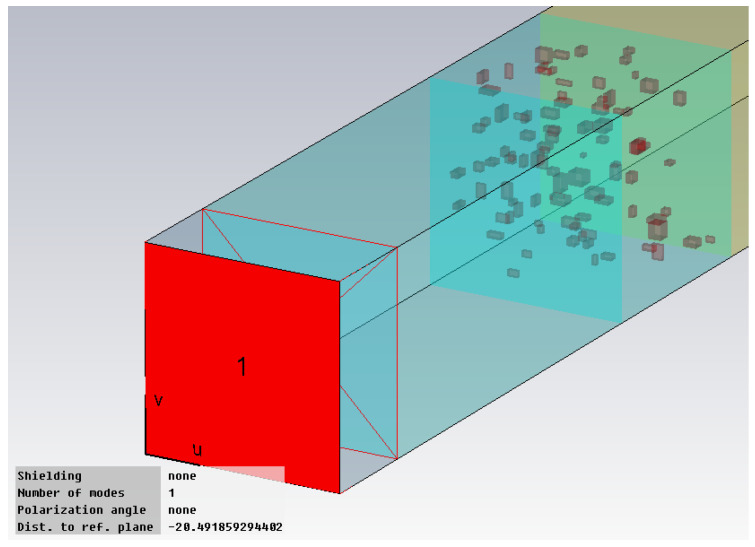
Setting the conditions at the input station.

The hot melt was considered with an addition of metal powders with the optimal concentration of 10% in mass, as experimentally demonstrated in [28], and with three particle sizes (1 μm, 5 μm, and 25 μm). The simulation presumed a theoretical scenario of uniform particle distribution within the polymer mass, which is technologically challenging as well as a more realistic technological scenario of random particle distribution, which has the disadvantage of being more difficult to implement in the simulation due to longer operating time. The modeling stages presumed the following steps: modeling of hot-melt interlayer (Figure 1); modeling of the junction between one substrate and hot-melt interlayer (Figure 2); modeling of the full structure of the bonded material (Figure 3); setting the conditions at the input station (Figure 4).

**Figure 5:**
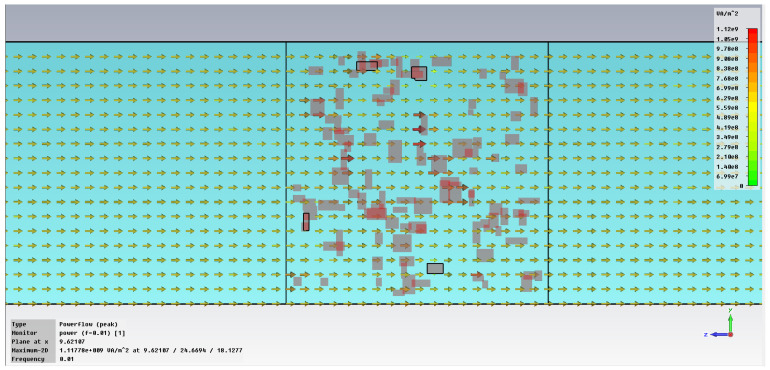
Power transfer, [VA/m^2^]: material 1—LDPE, hot melt of HDPE with inserts; material 2—LDPE, 0.01 GHz, dimension 1 μm.

**Figure 6:**
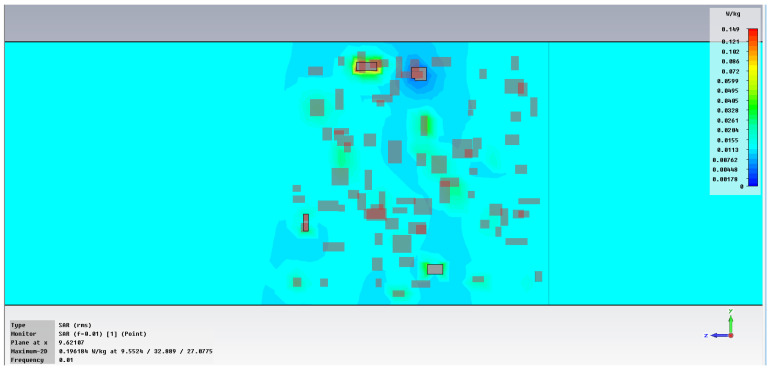
SAR, [W/m^3^]: material 1—LDPE, hot melt of HDPE with inserts; material 2—LDPE, 0.01 GHz, dimension 1 μm.

The subsequent phase pertains to the initial analysis of power transfer in the bonding structure, Figure 5, with increased emphasis on the energy absorbed in the hot melt/SAR, Figure 6. In the demonstration case shown in Figure 5, it is clear that the metal inserts hinder the unrestricted flow of the electric field, with each blockage creating a potential hotspot for energy accumulation that can heat the hot melt with the metallic inserts. Figure 6 clearly indicates how individual metallic particles heat up and how energy accumulates around those particles, eventually dispersing throughout the volume of the hot melt.

**Figure 7:**
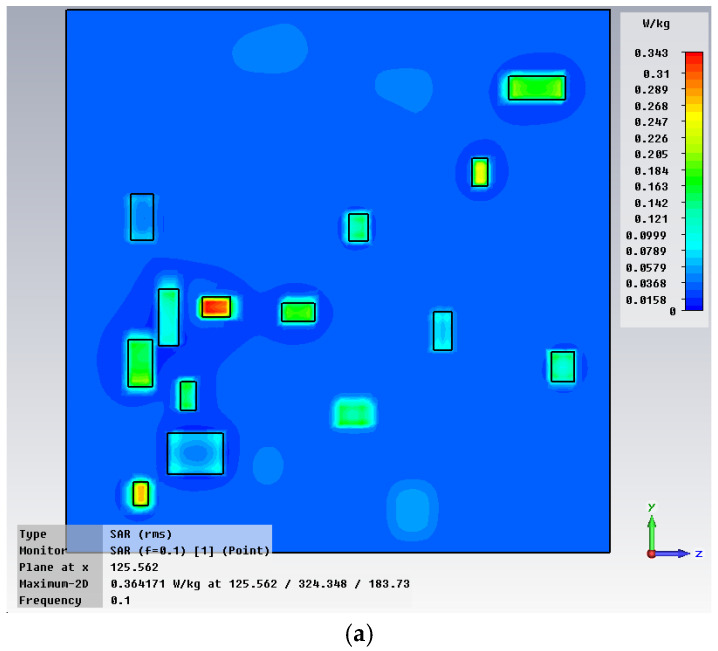

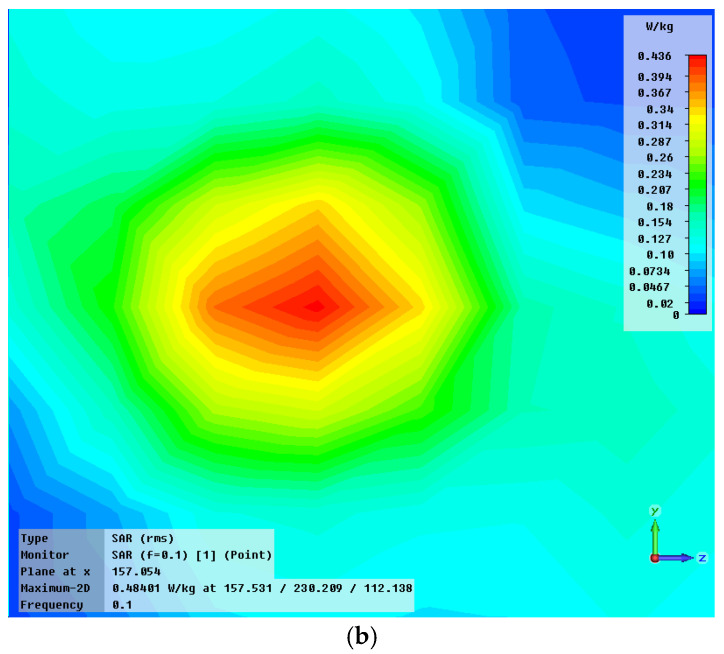
Max-point SAR: random particle distribution in composite hot melt (**a**), and detail around an iron insert (**b**).

The example in Figure 7 shows the situation encountered in the case of random particle distribution, the case of 10% concentration of iron insertions with the dimension of 25 μm at a frequency of 0.1 GHz. It is observed that the presence of metallic inserts characterized by high losses has the effect of creating “centers” of electromagnetic energy dissipation, where an increase in the concentration of electromagnetic energy around the inserts is achieved. Due to the higher dielectric characteristics of the material corresponding to iron inserts, Figure 7, especially in the plane of the inserts, we encounter an increased energy dissipation, i.e., of 0.484 W/kg around the particles, compared to the average value of 0.364 W/kg within the composite hot melt.

The last stage of the simulation comparisons initially focused on the changes in the parameters: Total SAR, Max-point SAR, and Max-point SAR/Total SAR for all examined variants. Ultimately, the configurations of functional bonding combinations, employing hot-melt composites with metallic additives like Polyolefin–Polyolefin and Polyolefin–Al, were considered, examining the changes in SAR parameters in relation to frequency, across the most relevant spectrum for the technological bonding under electromagnetic fields, of 0.1–10 GHz.

## 3. Results and Discussion

### 3.1. Influence of Metallic Particles Size upon SAR Parameters of Hot Melts

In the following figures, the iron insertions are identified as Type A and the aluminum insertions as Type B.

**Figure 8:**
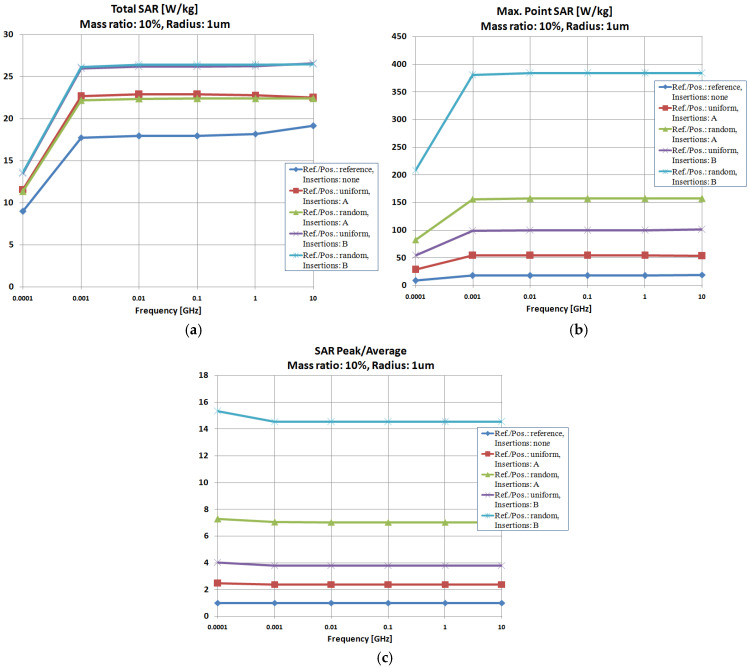
Investigation of insertion effect (type/dispersion) upon SAR parameters, size 1 μm: (**a**) Total SAR; (**b**) Max-point SAR; (**c**) SAR-Peak/SAR-Average.

**Figure 9:**
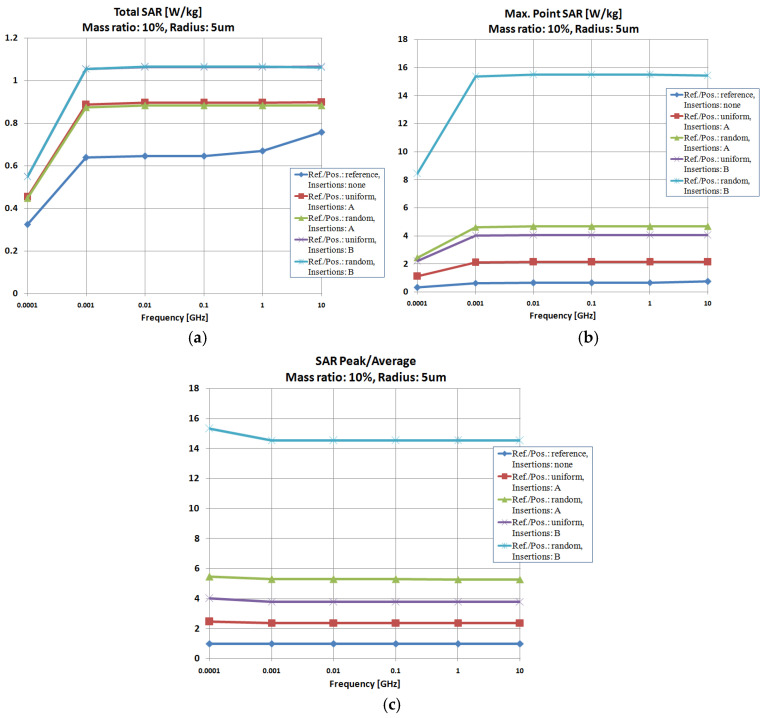
Investigation of insertion effect (type/dispersion) upon SAR parameters, size 5 μm: (**a**) Total SAR; (**b**) Max-point SAR; (**c**) SAR-Peak/SAR-Average.

**Figure 10:**
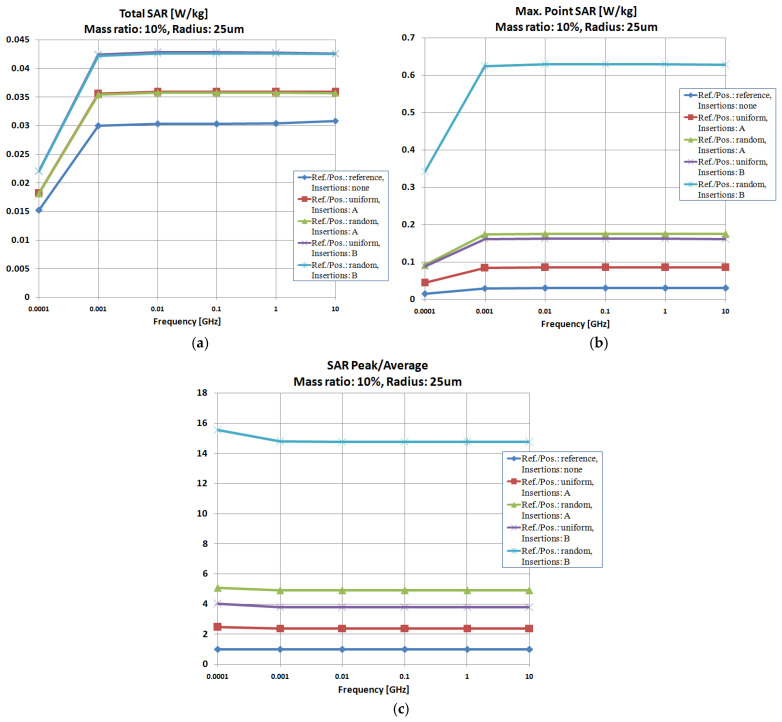
Investigation of insertion effect (type/dispersion) upon SAR parameters, size 25 μm: (**a**) Total SAR; (**b**) Max-point SAR; (**c**) SAR-Peak/SAR-Average.

Figure 8, Figure 9 and Figure 10 display the SAR parameters for the exposure to electromagnetic fields across a broader frequency range (0.0001–10 GHz) of composite hot melts with particle sizes of 1 μm, 5 μm, and 25 μm in scenarios featuring both uniform and random particle distributions. The optimal concentration of particles was 10% in every instance, as demonstrated in prior research [28].

The assessment of Total SAR for particle size of 1 μm, as shown in Figure 8a, indicated that Al is the most effective particle, while the distribution of particles has a minimal effect. Upon examining the Max-point SAR, Figure 8b, it is clear that the randomly distributed Al particles provide the most effective structure for absorbing electromagnetic energy.

It was observed that, regardless of the particle size, Al remains the most efficient particle, while the particle distribution has a negligible impact on Total SAR values, as shown in Figure 9 and Figure 10a. Analyzing the Max-point SAR, Figure 9 and Figure 10b, the randomly arranged Al particles with dimensions of 5 and 25 μm create the most efficient configuration for attaining the highest Max-point SAR values, similar to the results from Figure 8b.

However, distinct variations arise when analyzing the SAR parameter values for varying particle sizes. When considering only the Total SAR values in Figure 8a, Figure 9a and Figure 10a, it is clear that the smallest particles yield the most favorable outcomes, with Total SAR values decreasing from 26 W/kg at 1 μm to 1.05 W/kg at 5 μm and, ultimately, to 0.043 W/kg at 25 μm. A comparable trend is observed for Max-point SAR values, suggesting that at GHz frequency range, particles measuring 1 μm should be recommended. The upcoming simulations will consider these findings.

Concerning the SAR-Peak/SAR-Average values, the traits associated with Figure 8c, Figure 9c and Figure 10c are alike, verifying the findings regarding the effectiveness of Al particles dispersed randomly; in this case, the obtained values are nearly identical, irrespective of the particles’ sizes, which validates the energy balance illustrated, e.g., in Figure 6, concerning the report between the absorbed and transferred energy.

When examining the progression of SAR parameters with the electromagnetic field’s applied frequency, a continuous increase is observed relative to frequency, with significant results commencing from 0.01 GHz. The most beneficial effect of the inserts on the absorption capacity of the hot-melt material can be observed mainly for a particle size of 1 μm, as illustrated in Figure 8a, where the optimal frequency range for Total SAR is identified as 0.1–10 GHz, with the highest values nearing 10 GHz.

### 3.2. Energy Transfer Analysis vs. Frequency in Bonding Configurations

The example described below for the bonding configuration considered was the adhesion of an LDPE substrate to Al, utilizing a hot melt based on HDPE with Al inserts measuring 1 μm, subjected to various frequencies in the GHz range.

The simulation of energy transfer in bonding configurations is presented in Figure 11 and Figure 12 for the Power transfer [VA/m^2^], in Figure 13, Figure 14, Figure 15 and Figure 16 for Power loss density [W/m^3^], and, respectively, in Figure 17, Figure 18, Figure 19 and Figure 20 for SAR [W/kg].

**Figure 11:**
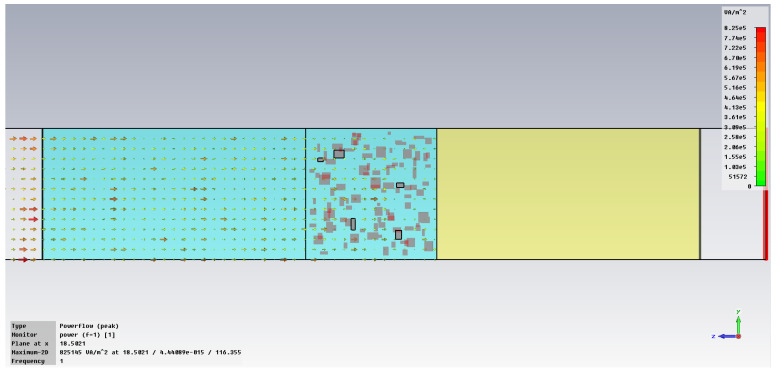
Power transfer, [VA/m^2^]: material 1—LDPE, HDPE adhesive with inserts; material 2—Al, 10 GHz.

**Figure 12:**
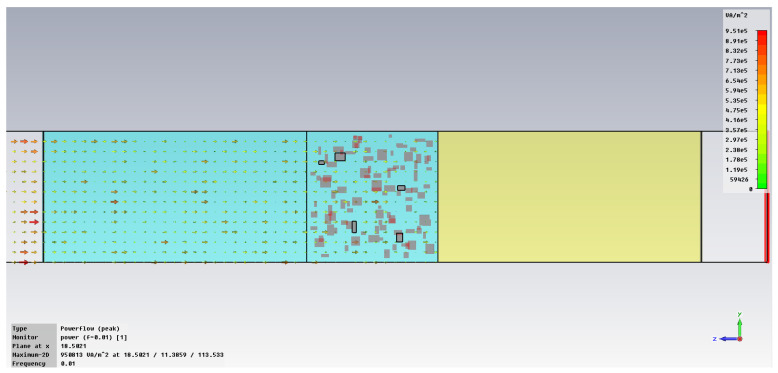
Power transfer, [VA/m^2^]: material 1—LDPE, HDPE adhesive with inserts; material 2—Al, 1 GHz.

The power transfer is clearly frequency-dependent, being approximately 16% higher at 1 GHz compared to 10 GHz, Figure 12 vs. Figure 11. This finding reinforces the point previously noted in Figure 8a, indicating that the greater the frequency, the more effectively the energy is absorbed in comparison to the energy transferred via the hot-melt material.

The subsequent research examines Power loss density and SAR across a wider frequency range of 0.01–10 GHz to evaluate the impact of frequency on the absorbed energy within the hot-melt material.

The synthesized findings are displayed in Table 3.

The results in Table 3 emphasize that the accumulated energy, expressed both as Power loss density and SAR results, decreases by about 100 times when the frequency is reduced from 10 GHz to 1 GHz. For much lower frequencies, the absorbed energy presents very low values, tending to a saturation at frequencies below 0.1 GHz.

The simulation indicates that the most effective bonding of polyolefins to Al should occur in the microwave frequency range, as close to 10 GHz as possible, to attain the highest efficiency. This also positively affects the feasibility and productivity of the bonding process, meaning reduced exposure times and/or reduced exposure power of microwaves at elevated frequencies. The results of the simulation correspond with the trends in the GHz frequency domain observed in the experimental results found in [29,34], where comparable hot melts were examined for bonding polyolefins to polyolefins. However, when bonding polyolefins to aluminum, the power loss density and SAR values are at least 100 times greater compared to bonding polyolefins to polyolefins, attributed to the conductive properties of aluminum, which influences the retention of additional energy within the hot melt mass, likely because of the Salisbury screen effect given by the assembly [35]. This aspect, also shown in Figure 3, influences the boundary conditions of the simulated cell at the input station, Figure 4, where the presence of Al results in a decreased penetration depth for the applied electromagnetic field.

### 3.3. Analysis of Microwave Bonding Efficiency

**Figure 21:**
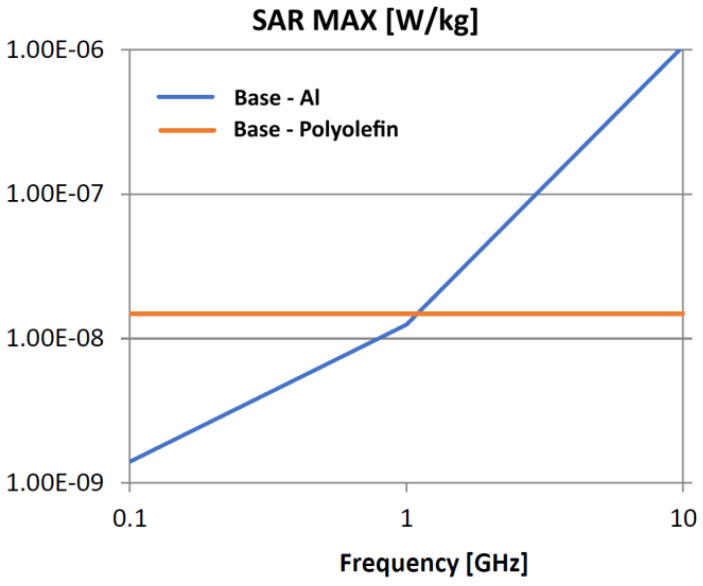
SAR MAX, [W/kg]: material—1 LDPE, LDPE adhesive; material 2—LDPE/Al.

**Figure 22:**
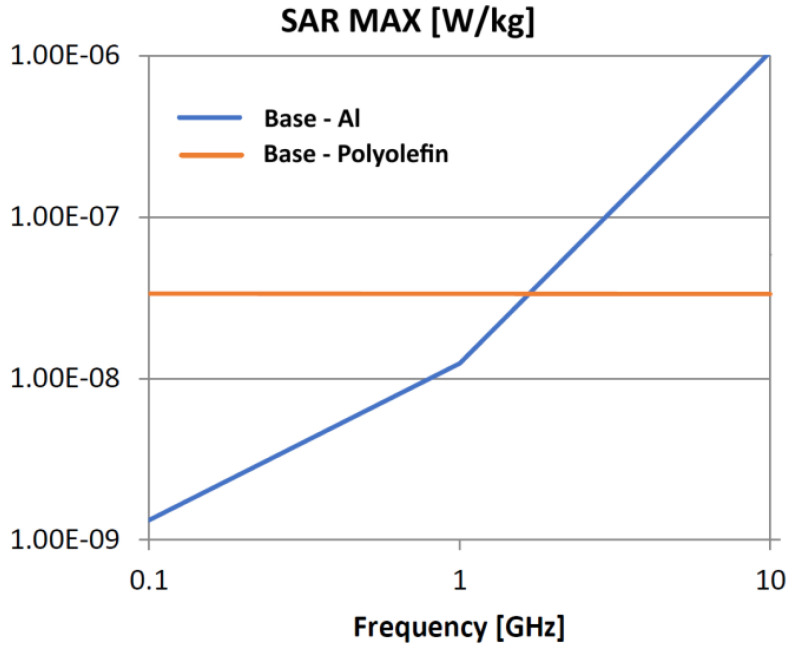
SAR MAX, [W/kg]: material—1 LDPE, LDPE adhesive; material 2—HDPE/Al.

**Figure 23:**
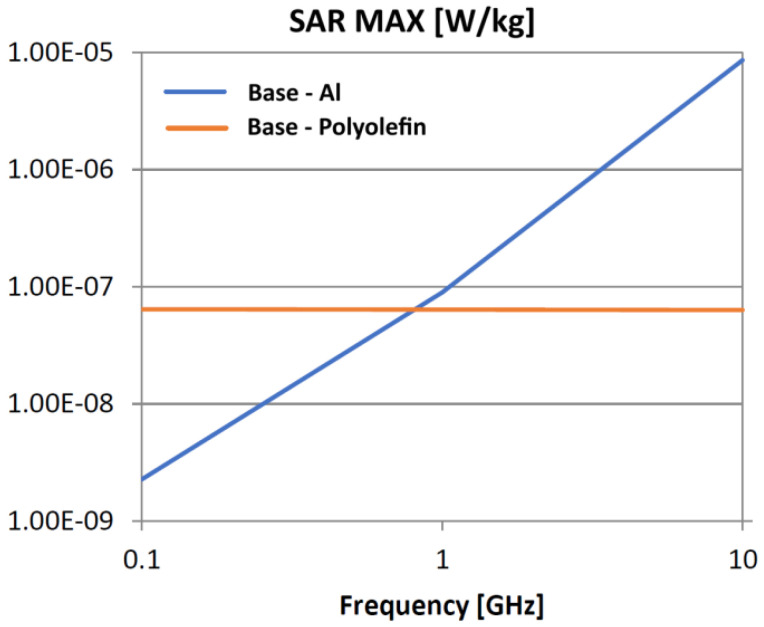
SAR MAX, [W/kg]: material—1 PP, LDPE adhesive; material 2—PP/Al.

**Figure 24:**
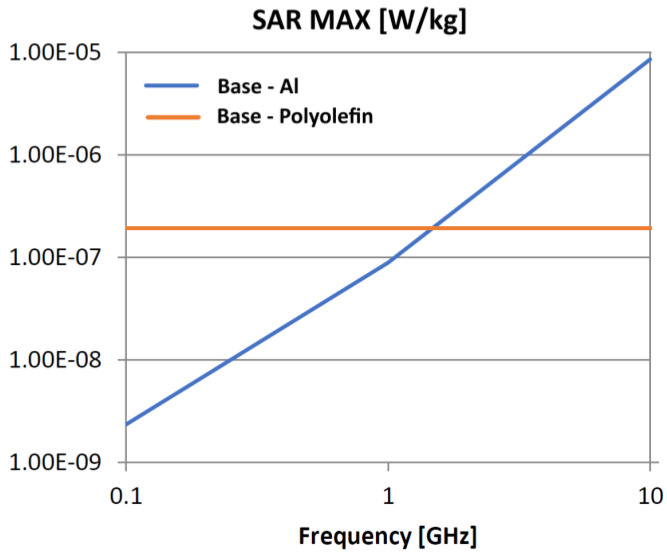
SAR MAX, [W/kg]: material—1 PP, LDPE adhesive; material 2—HDPE/Al.

**Figure 25:**
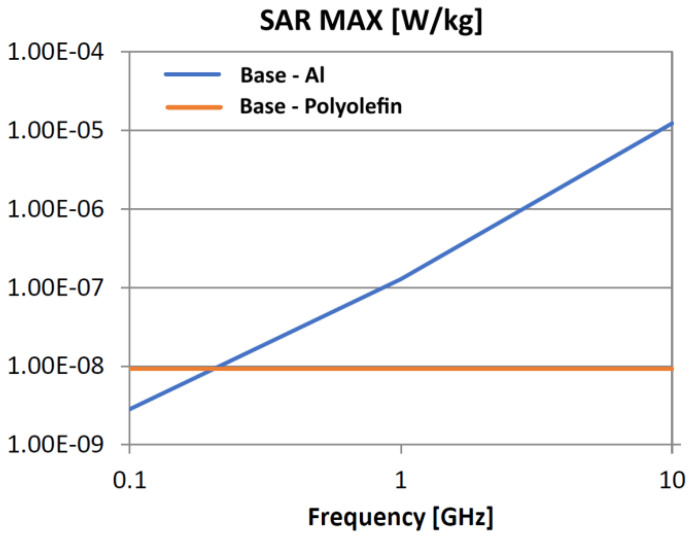
SAR MAX, [W/kg]: material—1 LDPE, HDPE adhesive; material 2—LDPE/Al.

**Figure 26:**
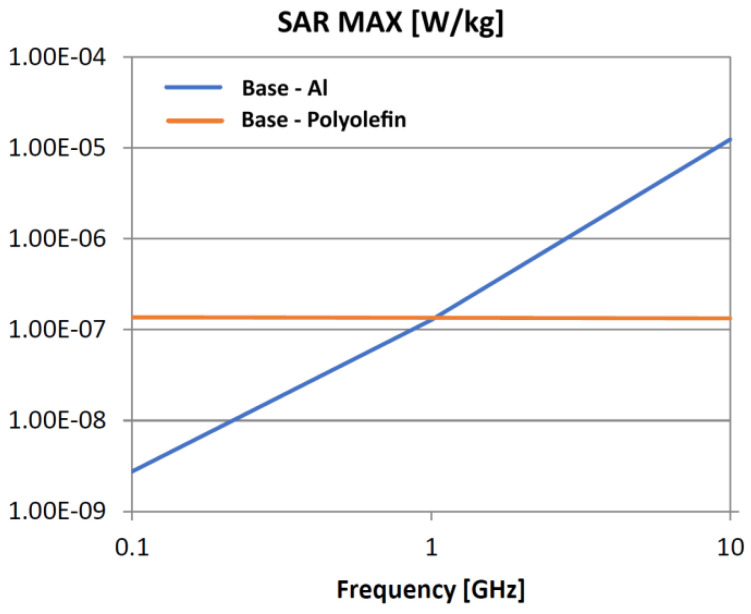
SAR MAX, [W/kg]: material—1 LDPE, HDPE adhesive; material 2—PP/Al.

**Figure 27:**
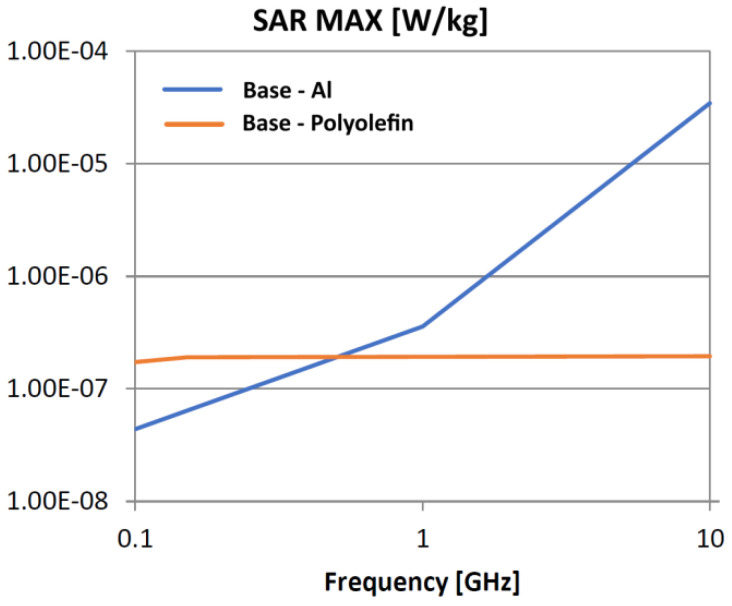
SAR MAX, [W/kg]: material—1 HDPE, HDPE adhesive; material 2—LDPE/Al.

**Figure 28:**
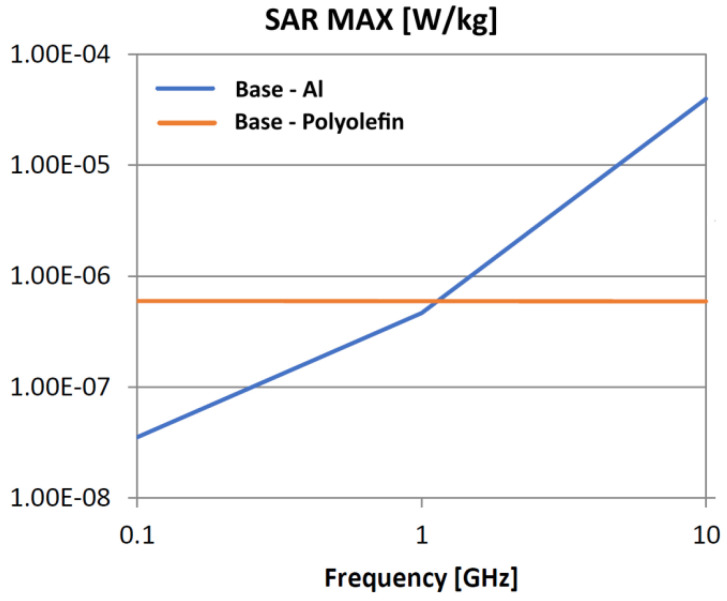
SAR MAX, [W/kg]: material—1 HDPE, HDPE adhesive; material 2—HDPE/Al.

**Figure 29:**
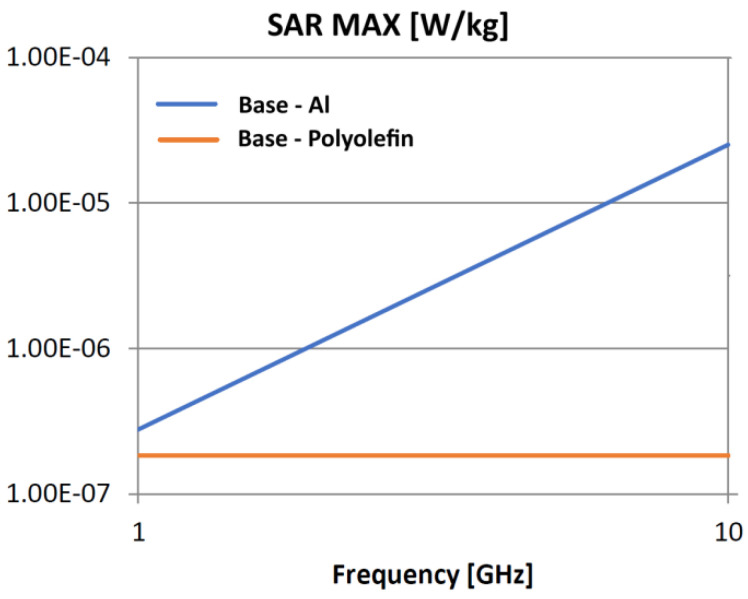
SAR MAX, [W/kg]: material—1 HDPE, PP adhesive; material 2—PP/Al.

**Figure 30:**
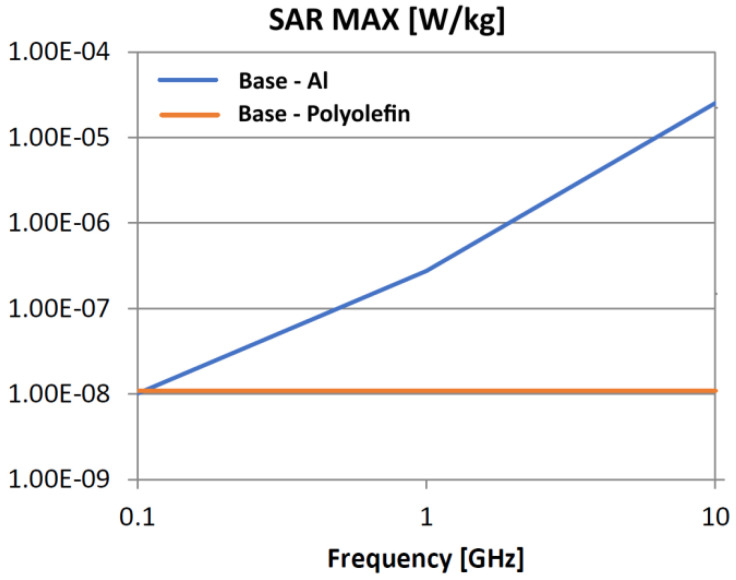
SAR MAX, [W/kg]: material—1 PP, PP adhesive; material 2—HDPE/Al.

**Figure 31:**
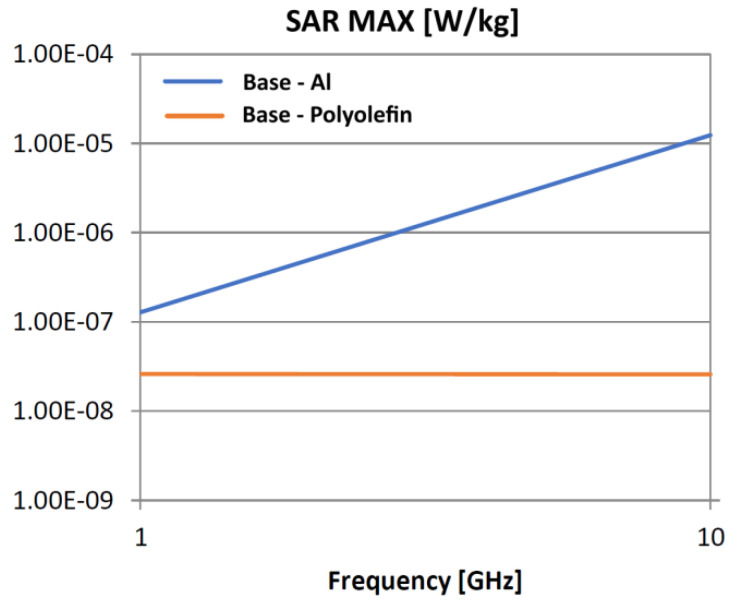
SAR MAX, [W/kg]: material—1 LDPE, PP adhesive; material 2—HDPE/Al.

The following analysis compares the microwave bonding effectiveness of polyolefins to polyolefins and to aluminum using various matrix-based hot melts, Figure 21, Figure 22, Figure 23, Figure 24, Figure 25, Figure 26, Figure 27, Figure 28, Figure 29, Figure 30 and Figure 31 (by base-polyolefin, each image refers to type of material 2). Under all models, the hot melts included a 10% incorporation of 1 μm Al particle size. The frequency range of 0.1–10 GHz was chosen, a designated range for thermo-activatable hot-melt adhesives.

Thereby, the previously stated hypotheses were validated, e.g., the results in Figure 27, where the evolution of SAR values vs. frequency for bonding HDPE to Al by using HDPE-based hot melt are presented, are in line with the results in Table 3. Conversely, the findings presented in Figure 21 and Figure 22 are partially validated by the experimental outcomes shown in [28] at 1 GHz, thus utilizing LDPE-based hot melts with a 7.5% inclusion of 800 nm Al particle size for polyolefins bonding, given that the size and concentration variations in particles in both scenarios are similar for an appropriate analysis.

Concerning the bonding of polyolefins, in all cases presented in Figure 21, Figure 22, Figure 23, Figure 24, Figure 25, Figure 26, Figure 27, Figure 28, Figure 29, Figure 30 and Figure 31, it indicates roughly similar and quasi-constant SAR values in the frequency domain, between 10^−8^ and 10^−7^ [W/kg]; however, for the bonding of polyolefins on Al, a significant rise is noted, with peak values approaching the 10 GHz threshold. If the recommended frequencies for hot-melt bonding of polyolefins were the approved technological frequencies of 0.915 GHz or 2.45 GHz [28,29], for bonding polyolefins to Al, the 2.45 GHz frequency is considered too low; perhaps, it would only be appropriate for hot melts made from HDPE or PP. For hot melts made from LDPE/HDPE, the frequency of 5.8 GHz would be suggested, which is a newly approved frequency used in innovative industrial applications to enhance heating efficiency in materials that poorly heat at lower frequencies.

It was also noted that, when joining polyolefins to aluminum, the power loss density and SAR values may be as much as 100 times higher than those for bonding polyolefins to polyolefins; some comparative results at 10 GHz are presented in Table 4.

The minimal difference of about 20 times was noticed for bonding LDPE to HDPE and, respectively, to Al, using LDPE-based hot melt, and the maximum difference of about 4000 times, in the case of joining PP to HDPE and, respectively, to Al, by using a PP-based hot melt. Relevant differences of about 1000 times were also found for the bonding configurations of LDPE to LDPE and, respectively, to Al, by using a HDPE-based hot melt, and for LDPE to HDPE and, respectively, to Al, with PP-based hot melt.

The simulation also highlighted slight variations in SAR values depending on the types of polyolefins or the matrix of the hot melts used, particularly when bonding polyolefins to polyolefins, with maximum values occurring, for instance, when HDPE serves as the substrate (till 4 times), or in the case of using the HDPE-based hot melt (till 150 times), as presented in Table 4.

Concerning the adhesion of polyolefins to Al, the hot melts derived from HDPE and PP result in elevated SAR values. Conversely, the SAR values are also elevated when bonding Al to HDPE. Thus, the optimal bonding of polyolefins to Al occurs when bonding HDPE to Al using HDPE-based hot melts.

The conclusions based on the simulation must be experimentally validated in relevant conditions, including the evaluation of the electromagnetic energy penetration and surface effect for larger thicknesses of materials. Conversely, the situations where elevated SAR values were observed concerning Al inserts resulted in improved efficiency of electromagnetic radiation absorption when binding polyolefins to Al might be generalized; a comparable evaluation should be conducted for other metal substrates that may need to be bonded to polyolefins for various industrial uses (e.g., polyolefins with copper or titanium in automotive parts or medical equipment). However, the simulation methodology introduced could bring significant advancement in assessing the effectiveness of bonding technology with dissimilar materials under electromagnetic energy by utilizing hot-melt adhesives containing metallic micro-additives, and it may be extended to determine the most efficient range of electromagnetic fields concerning energy or frequency.

## 4. Conclusions

This study presents the electromagnetic evaluation of hot-melt adhesives made from polyolefins with metallic ingredients for the bonding of polyolefins with aluminum. The research focused on determining the specific absorption rate for functional bonding combinations, such as Polyolefin–Polyolefin and Polyolefin–Al, examining the changes in electromagnetic parameters as a function of frequency from 0.1 to 10 GHz.

It was noted that, irrespective of the particle size, Al remains the most effective particle, whereas the particle distribution has a minimal effect on Total SAR values. The greatest positive impact of the inserts on the absorption ability of the hot-melt material is mainly seen with a particle size of 1 μm.

The simulation shows that optimal bonding of polyolefins to aluminum is expected ideally near 10 GHz, to achieve maximum efficiency, as SAR results decreased by about 100 times when the frequency is reduced from 10 GHz to 1 GHz, i.e., from 25.1 to 0.259 × 10^−6^ [W/kg]. The use of a higher frequency beneficially influences the viability and efficiency of the bonding process, leading to decreased exposure durations and/or lowered power of microwaves. When joining polyolefins to aluminum, the power loss density and SAR values are at least 100 times higher than those for bonding polyolefins to polyolefins, e.g., even over 1000 times, from 10^−8^ to 10^−5^ [W/kg] in the case of joining PP to HDPE and, respectively, to Al, by using a PP-based hot melt, or LDPE to LDPE and, respectively, to Al, by using a HDPE-based hot melt, and for LDPE to HDPE and, respectively, to Al, with PP-based hot melt. A possible explanation is due to aluminum’s conductive characteristics, which affect the retention of extra energy in the hot melt mass, probably due to the Salisbury screen effect produced by the bonding assembly.

For hot melts created from polyethylene, a frequency of 5.8 GHz is recommended, a recently authorized frequency utilized in cutting-edge industrial applications. Regarding the bonding of polyolefins to Al, the hot melts obtained from HDPE and PP produce higher SAR values. On the other hand, the SAR values rise when aluminum is bonded to HDPE. Consequently, the best adhesion of polyolefins to Al happens when joining HDPE to Al with HDPE-based hot melts.

The proposed simulation approach has the potential to greatly enhance the evaluation of bonding technology with dissimilar materials under electromagnetic energy, using hot-melt adhesives with metallic micro/nano-additives, and it could be expanded to identify the optimal range of electromagnetic fields in terms of energy or frequency.

## Figures and Tables

**Figure 13:**
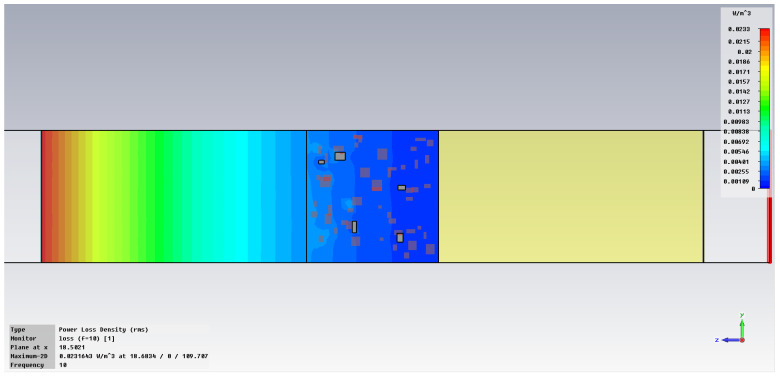
Power loss density, [W/m^3^]: material 1—LDPE, HDPE adhesive with inserts; material 2—Al, 10 GHz.

**Figure 14:**
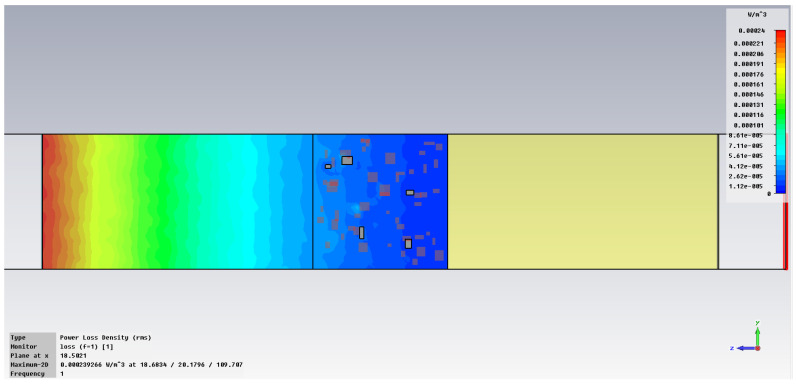
Power loss density, [W/m^3^]: material 1—LDPE, HDPE adhesive with inserts; material 2—Al, 1 GHz.

**Figure 15:**
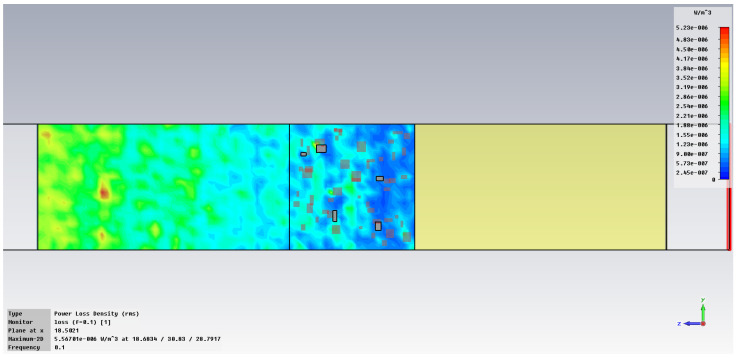
Power loss density, [W/m^3^]: material 1—LDPE, HDPE adhesive with inserts; material 2—Al, 0.1 GHz.

**Figure 16:**
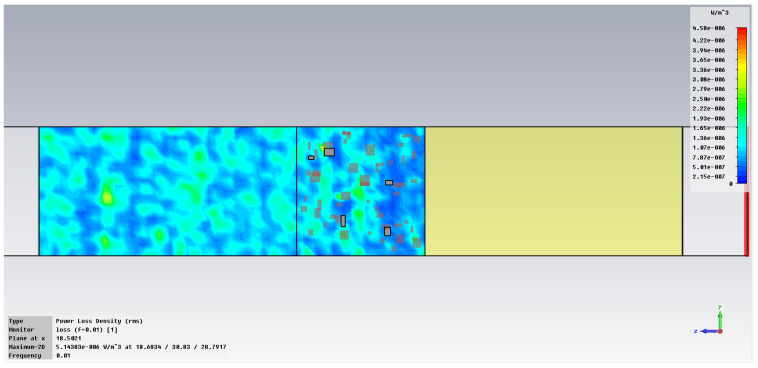
Power loss density, [W/m^3^]: material 1—LDPE, HDPE adhesive with inserts; material 2—Al, 0.01 GHz.

**Figure 17:**
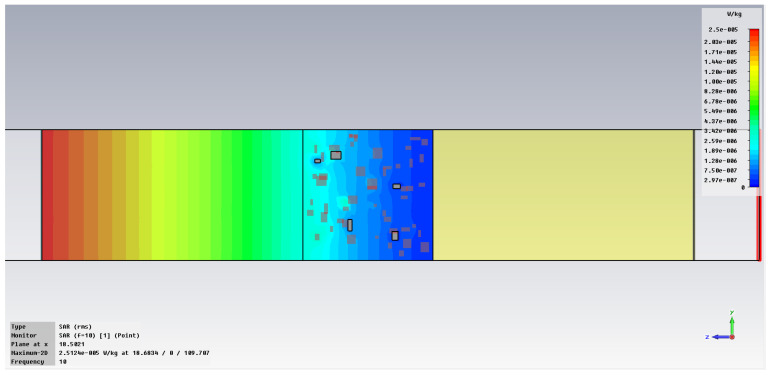
SAR, [W/kg]: material 1—LDPE, HDPE adhesive with inserts; material 2—Al, 10 GHz.

**Figure 18:**
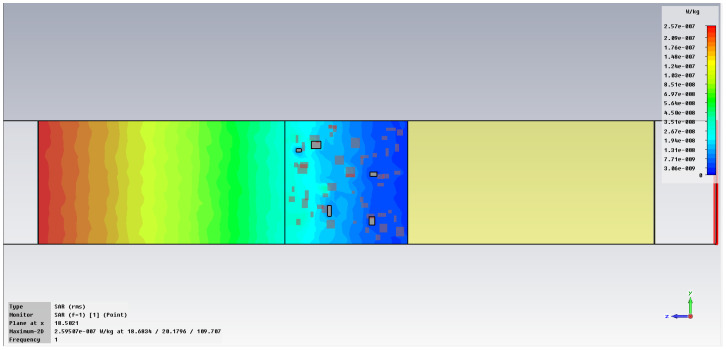
SAR, [W/kg]: material 1—LDPE, HDPE adhesive with inserts; material 2—Al, 1 GHz.

**Figure 19:**
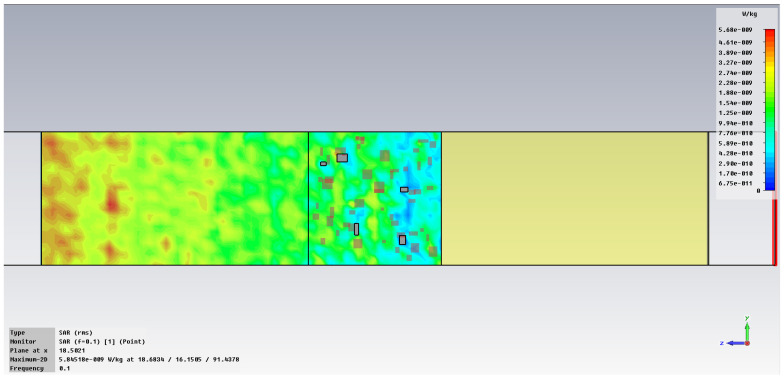
SAR, [W/kg]: material 1—LDPE, HDPE adhesive with inserts; material 2—Al, 0.1 GHz.

**Figure 20:**
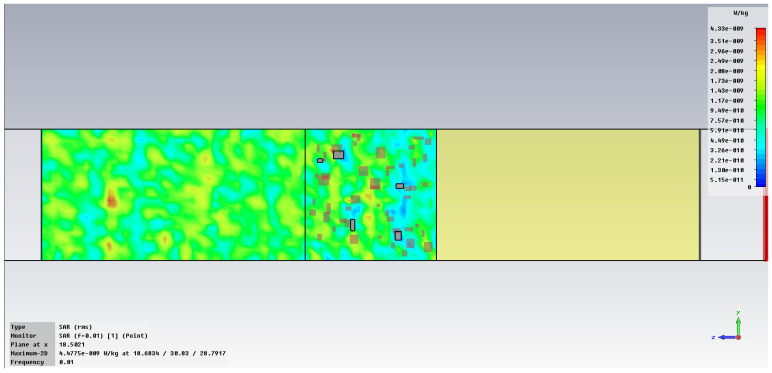
SAR, [W/kg]: material—1 LDPE, HDPE adhesive with inserts; material 2—Al, 0.01 GHz.

**Table 1:** Polyolefins model parameters.

Matrix	LDPE	HDPE	PP
Model type	Normal	Normal	Normal
Thermal conductivity	0.48 [W/K/m]	0.48 [W/K/m]	0.29 [W/K/m]
Density	945 kg/m^3^	945 kg/m^3^	905 kg/m^3^
Electrical permittivity ε_∞_	2.2	2.20	2.31
Electrical permittivity ε_S_	4	4	4
Relaxation time	0.2 s	0.2 s	0.2 s
Magnetic permeability	1	1	1
Specific heat	1.9 kJ/K/kg	1.9 kJ/K/kg	1.8 kJ/K/kg

**Table 2:** Metallic particles model parameters.

Material	Fe	Al
Model	Lossy metal	Lossy metal
Electrical conductivity	1.04 × 10^7^ [S/m]	3.56 × 10^7^ [S/m]
Thermal conductivity	79.5 [W/K/m]	237.0 [W/K/m]
Density	7870.0 kg/m^3^	2700.0 kg/m^3^
Specific heat	0.45 kJ/K/kg	0.9 kJ/K/kg

**Table 3:** Comparative Power loss density and SAR results vs. frequency.

Frequency [GHz]	Power Loss Density, [W/m^3^] × 10^−4^	SAR [W/kg] × 10^−6^
10	232	25.1
1	2.39	0.259
0.1	0.0556	0.00584
0.01	0.0514	0.00448

**Table 4:** Comparative SAR results vs. frequency for different bonding configurations.

Bonding Configuration	SAR [W/kg] × 10^−8^
LDPE to LDPE/Al, LDPE-based hot melt	1.15/100
LDPE to HDPE/Al, LDPE-based hot melt	5.5/100
PP to PP/Al, LDPE-based hot melt	8/950
PP to HDPE/Al, LDPE-based hot melt	20/950
LDPE to LDPE/Al, HDPE-based hot melt	1/1050
LDPE to PP/Al, HDPE-based hot melt	150/1050
HDPE to LDPE/Al, HDPE-based hot melt	25/6000
HDPE to HDPE/Al, HDPE-based hot melt	800/6000
PP to HDPE/Al, PP-based hot melt	1/4300
LDPE to HDPE/Al, PP-based hot melt	4/1050

## Data Availability

The original contributions presented in this study are included in the article. Further inquiries can be directed to the corresponding author.
